# Bone and muscle crosstalk in steroid-induced osteonecrosis of the femoral head: a four-axis pathophysiological framework

**DOI:** 10.3389/fimmu.2026.1782149

**Published:** 2026-04-01

**Authors:** Yan Yan, Jiawen Zhang, Nongyi Li, Junjie Li, Yishu Wen, Yi Wang, Yingru Huang

**Affiliations:** 1 School of Acupuncture-Moxibustion and Tuina, Chongqing University of Chinese Medicine, Chongqing, China; 2 The Third Affiliated Hospital of Beijing University of Chinese Medicine, Beijing, China

**Keywords:** bone-muscle comorbidity, bone-muscle crosstalk, pathological mechanisms, teroid-induced osteonecrosis of the femoral head, translational medicine

## Abstract

Steroid-induced osteonecrosis of the femoral head (SONFH) is a challenging orthopedic disease worldwide. Previous research has long focused on bone structure repair; however, bone and muscle are now recognized as functionally interconnected units coupled through biomechanics throughout the lifespan. Recent studies suggest that SONFH is not an isolated single-organ disorder but rather aligns with a systemic comorbid state characterized by the synergistic decline of bone and muscle function. Understanding the pathophysiology of bone and muscle crosstalk in SONFH is essential for its prevention and treatment. In this review, we propose an integrated pathological framework—the four-axis pathological mechanism of bone and muscle crosstalk in SONFH. We elaborate in detail on the mechanisms of bone and muscle crosstalk along four pathological axes—blood supply, lipid metabolism homeostasis, inflammation–immune regulation, and mechanical transduction—as well as the cross-tissue signaling-mediated synergistic damage among these axes: Imbalance in the blood supply axis may contribute to parallel ischemia in bone and muscle via shared pathways such as decreased HIF-1α/VEGF and inhibited NO/eNOS, which has been associated with endothelial dysfunction and impaired angiogenesis; Dysregulation of the lipid metabolism axis promotes bone marrow adiposity and muscular lipid accumulation by modulating key factors such as PPARγ and PGC-1α, as well as signaling pathways including PI3K/Akt/mTOR; Activation of the inflammation–immune axis exacerbates bone resorption and muscle atrophy through pathways such as NF-κB and STAT3, along with imbalanced immune cell polarization; Abnormalities in the mechanical axis create a vicious cycle of bone–muscle co-deterioration due to reduced bone load-bearing capacity and diminished muscular support function. This review further highlights current research gaps, including the insufficient systematic analysis of multi-axis interactive mechanisms, the lack of in-depth verification of bone-muscle crosstalk via multi-dimensional technologies, and the limited research on multi-target combined interventions targeting the bone-muscle unit. It proposes that future studies should strengthen systematic investigation into the interactive mechanisms among multiple pathological axes and develop combined intervention strategies targeting both bone and muscle. This will provide important insights for establishing an integrated diagnostic and therapeutic model addressing both structure and function, as well as for developing future hip-preserving treatment strategies for SONFH.

## 1 Introduction

Long-term or high-dose glucocorticoids (GCs) administration is recognized as one of the most common predisposing factors for non-traumatic osteonecrosis of the femoral head (NONFH) (1), frequently observed in patients with autoimmune diseases (2), malignancies (3), and organ transplantation (4). Compared with the general population, individuals subjected to GC therapy exhibit a significantly elevated incidence of NONFH, ranging from approximately 3% to 40% (5). Although hip-preserving strategies continue to evolve (6), natural history studies indicate that approximately 60% of cases eventually progress to collapse (7), and about 70%–80% undergo total hip arthroplasty (THA) (8). Previous research paradigms have long focused on alterations in bone structure (9–11). However, emerging evidence suggests that a considerable proportion of patients exhibit abnormalities in gait parameters (12), as well as reduced muscle mass and increased fat infiltration (13), even before the occurrence of apparent radiographic collapse. These observations indicate that functional decline is not solely attributable to bone structural damage; rather, impaired muscle function and interactions within the bone–muscle unit also play critical roles. These clinical findings raise a pivotal question: muscle may not merely be a “victim” in SONFH, but may actively contribute to disease progression.

In the study of diseases such as osteoporosis (OP) (14), osteoarthritis (OA) (15), and lumbar disc herniation (LDH) (16), the bone and muscle crosstalk is increasingly being recognized as a novel systemic health model (17, 18). Bone and muscle are now regarded as a functionally interconnected organ pair, linked through biomechanical coupling across the lifespan (19). Bone and muscle exhibit a high degree of coupling in energy metabolism (20, 21) and mechanical load transmission (22). Impairment in the structure or function of either tissue may affect the other via inter-tissue signaling (23–25). Based on these findings, we propose that SONFH may not be merely localized osteonecrosis, but rather a systemic disease process involving the coordinated degeneration of both bone and muscle. However, whether in current guidelines (26), clinical pathways (27), or mechanistic studies (28), the research framework for SONFH remains centered on pathological changes in bone structure, and has not yet systematically incorporated factors such as peri−hip muscle status, muscle strength levels, or intramuscular fat infiltration into the disease assessment framework.

Therefore, this review proposes an integrated pathological framework: the four-axis mechanism of bone-muscle crosstalk in SONFH. This framework systematically elucidates how bone and muscle undergo synergistic damage through shared pathways and cross-tissue signaling under GC exposure, focusing on four pathological axes: blood supply, lipid metabolic homeostasis, inflammation–immune regulation, and mechanical transduction. Furthermore, it suggests potential future research directions and interdisciplinary approaches, thereby providing insights for developing integrated diagnostic and therapeutic strategies for SONFH that address both structural and functional aspects.

## 2 Imbalance of the blood supply axis: parallel ischemic mechanisms in osteonecrosis and muscle atrophy

### 2.1 Impairment of femoral head microcirculation induced by GCs

Blood supply impairment is widely regarded as one of the core initiating pathological mechanisms of SONFH (29). The femoral head receives its blood supply from the fan-shaped branches of the medial and lateral femoral circumflex arteries, which feature relatively sparse anastomoses and limited compensatory collateral circulation (30, 31), collectively resembling an end-artery supply pattern. This anatomical characteristic renders it highly sensitive to alterations in blood flow; even minor increases in vascular resistance or obstructions within the vascular pathways can induce local ischemia. Under conditions of long-term or high-dose GC exposure, vascular endothelial cell function is significantly compromised (32, 33). The underlying mechanism is associated with significant impairment of nitric oxide (NO) synthesis and endothelial nitric oxide synthase (eNOS) activity. NO is one of the key vasodilator produced by endothelial cells. GCs can reduce the bioavailability of vascular NO by suppressing the expression (34) or activity (35) of eNOS in vascular endothelial cells, or by increasing the production of reactive oxygen species (ROS) (36). This process may lead to diminished endothelium-dependent vasodilation and reduced vascular reactivity, which has been identified as a key contributor to decreased local blood perfusion in multiple pre-clinical SONFH models (37). Furthermore, GCs disrupt the balance between NO and vasoconstrictive factors such as endothelin-1 (ET-1), inhibiting NO generation while simultaneously promoting the expression of ET-1 and its receptors (38). This shifts overall vascular tone towards a constrictive state, further exacerbating local ischemia in the femoral head. In addition, studies have found that GCs can elevate the levels of plasminogen activator inhibitor-1 (PAI-1), a fibrinolysis inhibitor, thereby suppressing fibrinolytic system function (39, 40). This induces a hypercoagulable–hypofibrinolytic state, promoting the formation of microthrombi and increasing thrombus stability. These microthrombi further increase hemorheological resistance within the microvasculature, reducing the blood supply to the femoral head (29, 41). GCs exposure may also activate the coagulation cascade and upregulate the levels of various coagulation factors, including fibrinogen (42), further aggravating the risk of microthrombosis. The adverse effects of this coagulation–fibrinolysis imbalance are more pronounced in bone structures like the femoral head, which have limited collateral circulation (43, 44).

**Figure 1:**
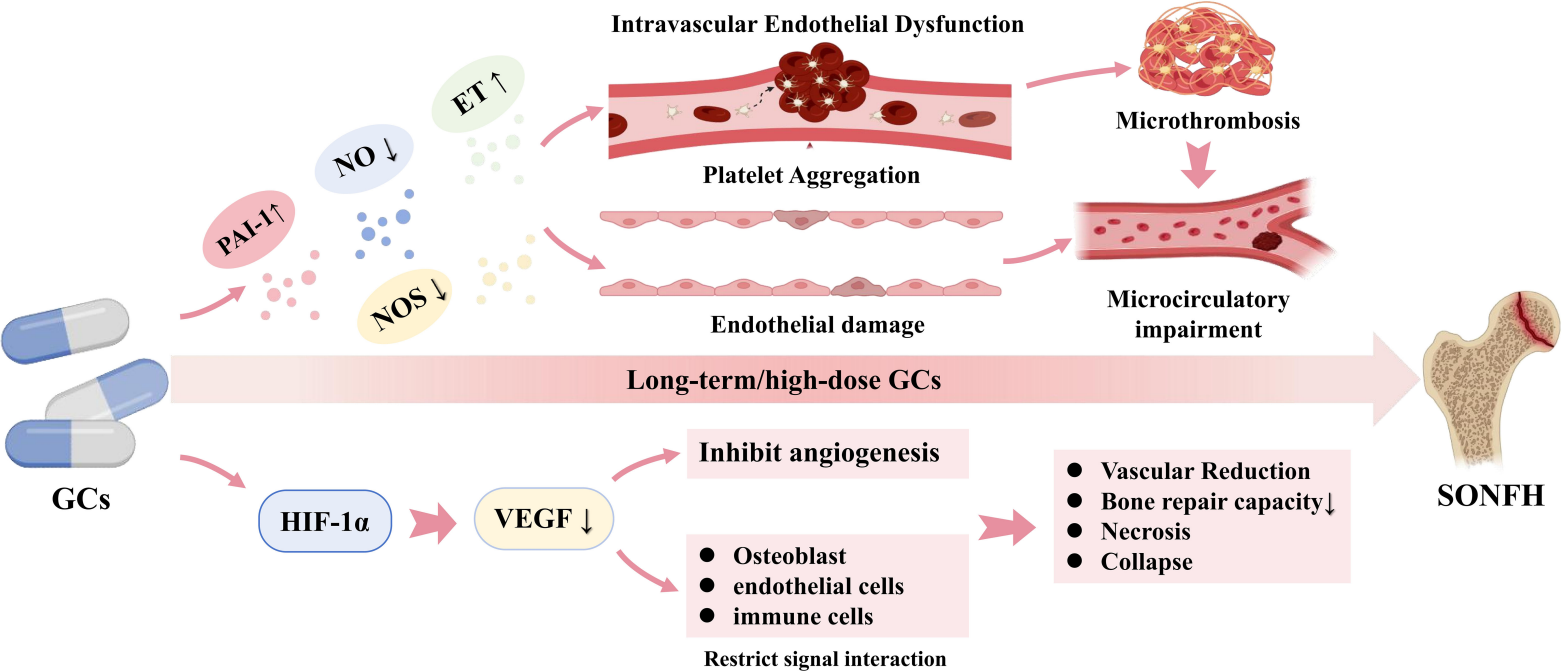
The pathological mechanisms of the blood supply axis within the bone microenvironment. Long-term/high-dose GCs induce intravascular endothelial dysfunction and microthrombosis, accompanied by the robust downregulation of HIF-1α and VEGF. These critical molecular alterations cause severe microcirculatory impairment, thereby restricting angiogenesis and diminishing bone repair capacity, ultimately precipitating SONFH. Solid arrows indicate direct molecular activation, cellular transformation, or pathological progression. Upward and downward arrows (↑/↓) denote the pathological upregulation or downregulation of specific targets, respectively.

Impaired angiogenesis is widely recognized as one of the key mechanisms underlying SONFH. Long-term or high-dose GCs administration can inhibit the formation of new blood vessels by suppressing the HIF-1α/VEGF signaling pathway (45). HIF-1α is one of the critical regulator of the cellular response to hypoxia (46). Under ischemic conditions, HIF-1α promotes the production of VEGF. The upregulated VEGF stimulates endothelial cell proliferation, increases vascular density in the necrotic area of the femoral head, and restores local blood supply, thereby facilitating the repair of the osteonecrotic region (47, 48). However, under GCs exposure, the stability of HIF-1α is reduced, leading to decreased expression and secretion of VEGF, which consequently contributes to the occurrence of femoral head necrosis and collapse (31). Vascular impairment not only restricts blood supply to the femoral head but also disrupts signaling interactions among osteoblasts, endothelial cells, and immune cells, resulting in diminished bone repair capacity. Therefore, compromised blood supply serves as both a significant trigger for osteonecrosis and a fundamental cause of impaired repair and disease progression (**Figure 1**).

### 2.2 Muscle ischemia and impairment induced by GCs

The decline in muscle function is also closely associated with restricted blood supply (49). Muscle possesses a relatively rich capillary network (50); however, it operates in a state of sustained high metabolism during exercise, load-bearing, and posture maintenance, rendering it highly dependent on microcirculatory perfusion and blood flow regulation (51, 52). Once capillary density decreases or endothelium-dependent vasodilation is impaired, local muscle function deteriorates even in the presence of patent major vessels (53). This manifests as reduced muscle endurance, delayed post-exercise recovery, and progressive loss of muscle strength (54). High-dose GCs can inhibit muscle capillary formation (55), potentially shifting the oxygen supply status of muscle tissue from diffusion-limited to perfusion-limited. Concurrently, GCs impair the efficiency of mitochondrial oxidative phosphorylation, leading to insufficient adenosine triphosphate(ATP) synthesis (56). Furthermore, as mentioned previously, GCs can upregulate the expression of vasoconstrictor factors and exacerbate a systemic hypercoagulable–hypofibrinolytic state. This combination can result in chronic, mild, sustained vasoconstriction and hypoperfusion within the skeletal muscle microcirculation. The ensuing capillary rarefaction (57) and a decreased capillary-to-muscle fiber ratio (58) contribute to atrophic changes in muscle at both structural and functional levels, as well as diminished contractile function of muscle fibers.

**Figure 2:**
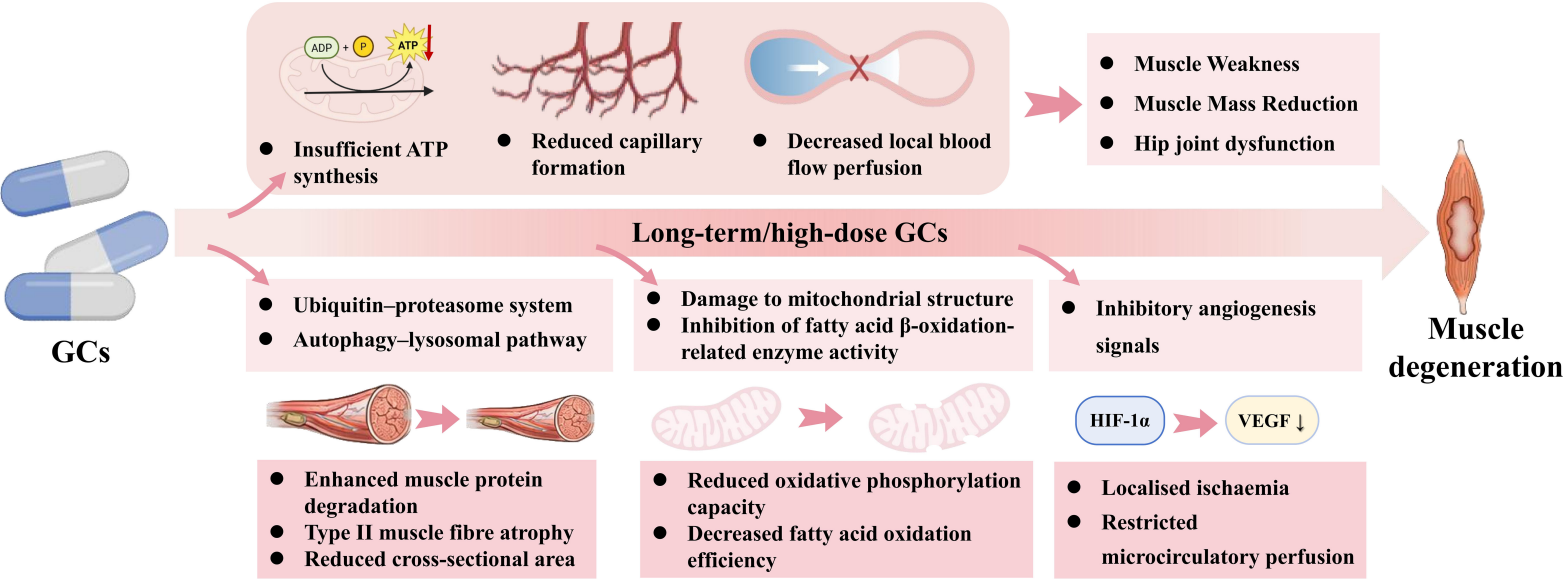
The pathological mechanisms of the blood supply axis within the muscle microenvironment. GCs induce localized ischemia, characterized by reduced capillary formation and profoundly decreased local blood flow perfusion. Concurrently, GCs inflict severe damage on mitochondrial structure and inhibit fatty acid β-oxidation, leading to insufficient ATP synthesis. These ischemic and metabolic deficits synergistically enhance muscle protein degradation via the ubiquitin-proteasome and autophagy-lysosomal pathways, driving type II muscle fiber atrophy and macroscopic muscle degeneration. Solid arrows indicate direct pathogenic progression. Upward and downward arrows (↑/↓) denote upregulation or downregulation of specific targets, respectively.

In addition to direct damage to the microvasculature, studies have shown that GCs can enhance muscle protein degradation by activating the ubiquitin-proteasome system and the autophagy-lysosome pathway (59), which may drive atrophy and a reduction in the cross-sectional area, primarily in type II muscle fibers (60). Concurrently, GCs impair the ultrastructure of muscle mitochondria and inhibit mitochondrial biogenesis, resulting in decreased oxidative phosphorylation capacity (61, 62). They also suppress the activity of enzymes involved in fatty acid β-oxidation, thereby reducing fatty acid oxidation efficiency (63). Under these conditions, even if the residual blood supply barely meets the basic metabolic demands at resting state, the muscle becomes inefficient at utilizing the limited oxygen and substrates during activity or loading, manifesting a dual deficiency of both low perfusion and low utilization. Although local ischemia and hypoxia can induce the activation of hypoxia-responsive pathways such as HIF-1α (47), in the context of GC-mediated suppression of pro-angiogenic signals, this compensatory response often fails to translate into effective vascular remodeling and microcirculatory improvement. This ultimately drives the progression of muscle from reversible dysfunction to structural atrophy. Notably, existing imaging studies and case reports indicate that in some patients with non-traumatic, particularly steroid-associated osteonecrosis of the femoral head, changes such as reduced cross-sectional area, increased fat infiltration, and weakened muscle strength are already present in the peri−hip musculature on the affected side, even before evident radiographic collapse occurs (13, 64). These muscular alterations show a certain correlation with the degree of local tissue ischemia, suggesting that the muscle changes may not be solely attributable to disuse but may involve an independent or parallel mechanism of ischemic injury (**Figure 2**).

### 2.3 Shared ischemic signaling pathways and axial linkage in bone and muscle

Accumulating evidence indicates that osteonecrosis and muscle atrophy are not merely two independent target organ injuries, but may be partially overlapping tissue phenotypes driven by the same axis of “impaired blood supply” under GC exposure. Their upstream driving factors are primarily concentrated in shared ischemic signaling pathways. First, systemic endothelial dysfunction, characterized by NO/eNOS inhibition and ET-1 upregulation, simultaneously places the microcirculation of multiple tissues, including the terminal arterial branches of the femoral head and the muscle capillary bed, in a state of high resistance and low perfusion. Second, the inhibition of the HIF-1α/VEGF axis and the consequent insufficient angiogenesis and microvascular rarefaction transform ischemia from a transient functional impairment into a persistent, structural blood flow deficiency. Furthermore, the ischemic state can further induce oxidative stress and inflammatory responses, forming a cross-tissue injury amplification effect.

Thus, under long-term or high-dose GCs exposure, the ischemic processes in the bone and muscle of SONFH do not occur in isolation. Instead, they share key signaling pathways, and their injuries possess characteristics of mutual amplification. As ischemia persists, cross-amplification between the two occurs through common pathways such as decreased HIF-1α/VEGF and NO/eNOS inhibition, which may drive an exponential acceleration in the deterioration of the local microenvironment. This manifests as decreased bone perfusion in the femoral head, affecting the metabolism of surrounding muscle groups, while damage to the peri-hip muscles further impedes blood circulation and venous return, perpetuating bone ischemia. This suggests that the blood supply axis is not only the pathological starting point of SONFH but also one of the core common driving forces behind bone and muscle functional degeneration. The imbalance of the blood supply axis is one of the earliest and most widespread pathological axes in SONFH, initiating not only the osteonecrosis process but also laying the foundation of energy and metabolic disorders for muscle function decline. Thus, within the bone-muscle crosstalk framework, the blood supply axis represents the pivotal axis of SONFH pathogenesis, functioning to synergistically aggravate the processes of the other pathological axes.

While most studies have separately investigated microcirculatory impairments in either the femoral head or skeletal muscle under GC exposure, few have utilized a unified *in vivo* model to directly evaluate the synchronous ischemic changes within the hip “bone-muscle unit.” Moreover, the causal relationship and temporal sequence between muscle ischemia and osteonecrosis remain to be elucidated. A classic sequential study in live mouse models by Weinstein et al (45) demonstrated that GCs initially trigger local ischemia and osteocyte apoptosis in the femoral head, processes that occur prior to bone mass loss and microstructural collapse. However, it remains unclear whether muscle microvascular impairment precedes femoral head ischemia or merely manifests as a secondary consequence of structural bone collapse. Furthermore, although molecules such as Sclerostin, Myostatin, and macrophage-derived exosomes (65, 66) are key mediators driving cross-tissue damage between bone and muscle, their precise mechanisms of action remain incompletely understood. This knowledge gap continues to hinder the development of targeted therapies aimed at simultaneously restoring microcirculation in both tissues.

## 3 Lipid metabolism axis: bone-muscle energy imbalance

### 3.1 Lipid metabolic disorders in bone tissue induced by glucocorticoids

Bone marrow adiposity is a notable alteration induced by lipid metabolism dysregulation in SONFH (67). Under the influence of GCs, the propensity of bone marrow mesenchymal stem cells (BMSCs) to differentiate into adipocytes is markedly enhanced (68, 69). This process is regulated by key transcription factors such as PPARγ and C/EBPα (70, 71). PPARγ, in particular, is considered one of the core factor promoting the adipogenic differentiation of BMSCs. Studies have shown that GCs can upregulate the expression of PPARγ, directing BMSCs more towards adipocyte differentiation (69). This leads to extensive hyperplasia and hypertrophy of adipocytes within the bone marrow cavity, thereby potentially compromising the blood supply to the femoral head. Secondly, lipid accumulation not only alters the composition of bone marrow adipocytes but may also interfere with fatty acid metabolic pathways within bone tissue through the release of free fatty acids, consequently inhibiting the utilization of fatty acids by osteoblasts. Existing research indicates that impaired fatty acid β−oxidation in osteoblasts can significantly weaken bone formation capacity (72, 73). Furthermore, bone marrow adipose tissue not only releases large amounts of fatty acids but can also influence osteocyte function through adipokines and metabolic mediators (74, 75). Additionally, lipid metabolism disorders induced by GCs can lead to insulin resistance, which has been linked to decreased levels of insulin-like growth factor-1 (IGF-1) (76). IGF−1 is a crucial regulator of osteoblast proliferation and differentiation, and its reduced levels significantly impair bone formation capacity.

**Figure 3:**
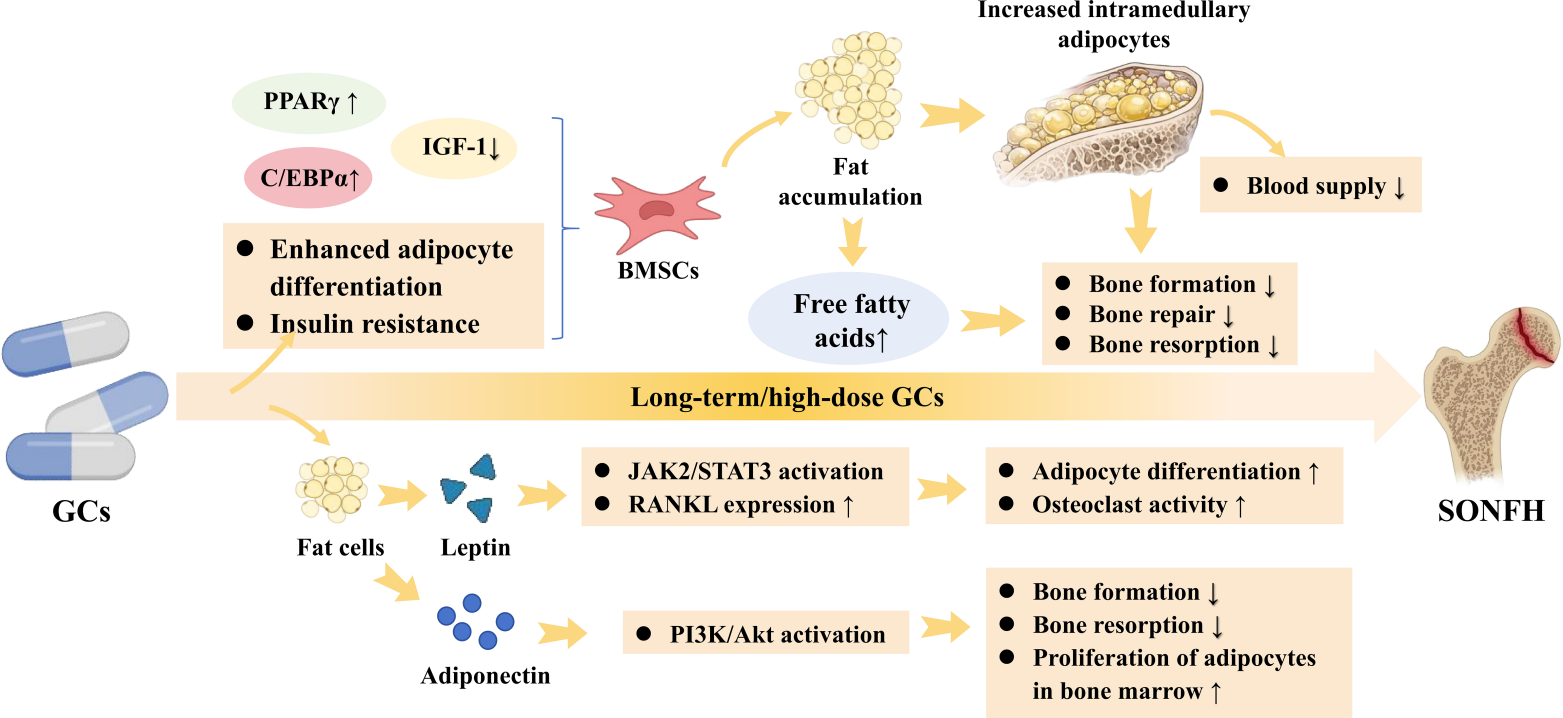
The pathological mechanisms of the lipid metabolism axis within the bone microenvironment. High-dose GCs potently upregulate PPARγ and C/EBPα while downregulating IGF-1, forcibly redirecting BMSCs towards adipogenic differentiation. The resulting intramedullary fat accumulation and elevated free fatty acids directly compromise local blood supply and bone formation. Simultaneously, adipocyte-derived leptin and adiponectin dysregulation activates the JAK2/STAT3 and PI3K/Akt pathways, upregulating RANKL expression and osteoclast activity, which actively accelerates SONFH progression. Solid arrows denote direct progression or molecular activation. Upward and downward arrows (↑/↓) indicate upregulation or downregulation.

Leptin and adiponectin are adipokines secreted by adipocytes. Leptin activates the JAK2/STAT3 pathway, leading to increased release of pro-inflammatory factors (77), promoting adipocyte differentiation and inhibiting osteogenic differentiation (78). Concurrently, low-dose leptin can also enhance osteoclast activity by promoting the expression of receptor activator of nuclear factor kappa-B ligand (RANKL) (79). Adiponectin regulates bone metabolism by activating the PI3K/Akt pathway (80). However, in SONFH, serum adiponectin levels are negatively correlated with bone loss, trabecular microstructure deterioration, and bone mineral density (81). This phenomenon may be related to abnormal fluctuations in adiponectin levels, which could disrupt the bone-fat balance by inhibiting bone formation, promoting bone resorption, or by enhancing the hyperplasia and hypertrophy of adipocytes within the bone marrow (**Figure 3**).

### 3.2 GC-induced dysregulation of lipid metabolism in muscle

**Figure 4:**
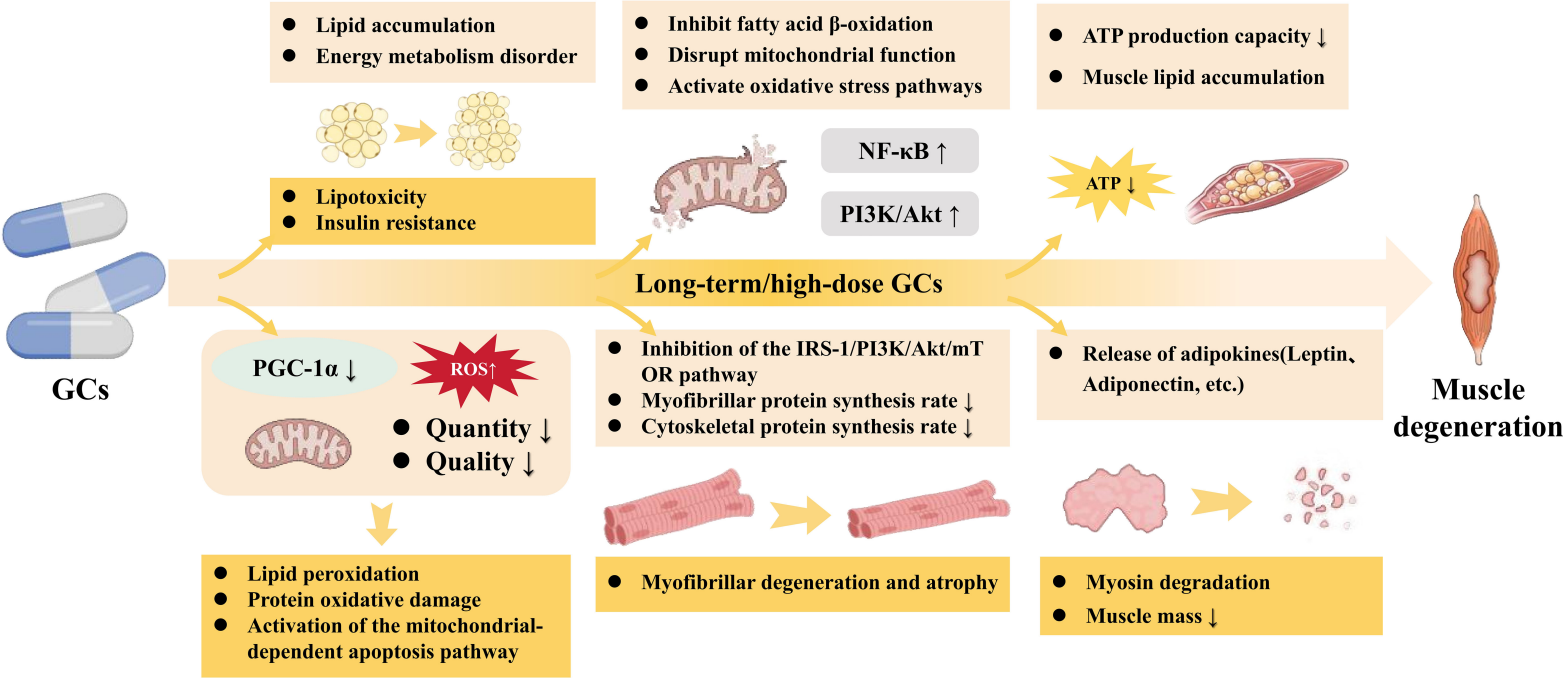
The pathological mechanisms of the lipid metabolism axis within the muscle microenvironment. GCs provoke severe muscular lipid accumulation and energy metabolism disorders. The subsequent lipotoxicity and ROS overproduction disrupt mitochondrial function and intensely activate oxidative stress pathways. Furthermore, GCs inhibit the IRS-1/PI3K/Akt/mTOR pathway, critically reducing myofibrillar protein synthesis, while activating NF-κB pathways to accelerate myosin degradation. Ultimately, this intense lipotoxicity precipitates rapid myofibrillar degeneration and muscle atrophy. Solid arrows indicate direct molecular activation or pathological progression. Upward and downward arrows (↑/↓) denote upregulation or downregulation.

Long-term or high-dose GCs exposure affects the process of lipid metabolism in muscle, leading to lipid accumulation and energy metabolism disorders. GCs act through their receptors in muscle to regulate fatty acid metabolic pathways, altering the balance between fatty acid oxidation and fatty acid synthesis, thereby affecting the acquisition and utilization of energy in muscle. Studies have shown that GCs can promote the accumulation of lipid droplets within muscle (63). This lipid accumulation is believed to potentially induce lipotoxic reactions by inhibiting fatty acid β−oxidation, impairing mitochondrial function, and activating oxidative stress pathways (82), thereby further damaging the metabolic homeostasis of myocytes. The sustained overload of excessive fatty acids on mitochondria, coupled with decreased oxidative phosphorylation efficiency, weakens ATP production capacity (83, 84). Furthermore, the expression of key regulators of mitochondrial biogenesis, such as peroxisome proliferator-activated receptor-γ coactivator-1α (PGC-1α), is downregulated (85), leading to a simultaneous decline in both mitochondrial quantity and quality reserves. Dysfunctional mitochondria produce excessive ROS, which in turn induce lipid peroxidation, protein/DNA oxidative damage, and activate the mitochondria-dependent apoptotic pathway (86), ultimately contributing to muscle fiber degeneration and atrophy. Lipotoxicity and insulin resistance can inhibit the activity of anabolic pathways within muscle, such as the IRS-1/PI3K/Akt/mTOR pathway, which may lead to a decreased synthesis rate of myofibrillar and cytoskeletal proteins (87, 88). Under the influence of GCs, adipokines form a complex regulatory network with muscle fat accumulation and energy metabolism imbalance. For instance, decreased adiponectin levels are often accompanied by GC-induced fatty acid accumulation and muscle atrophy, further exacerbating abnormal fatty acid oxidation and lipid metabolism disorders in muscle (89, 90) (**Figure 4**).

### 3.3 Shared pathways and axial connections in bone-muscle lipid metabolism dysregulation

As mentioned earlier, GC-induced lipid metabolism disorders do not occur in isolation at the bone and muscle ends but are established upon a relatively unified metabolic sensing and signal transduction network. When the body is under GCs exposure and systemic lipid load increases, these molecular axes exhibit highly isomorphic responses at the bone and muscle ends: the bone end manifests as upregulation of PPARγ, a shift of BMSCs towards the adipogenic lineage, and inhibition of osteogenesis-related PI3K/Akt/mTOR signaling; the muscle end manifests as downregulation of PGC-1α, restricted mitochondrial biogenesis, and weakened IRS-1/PI3K/Akt/mTOR-mediated protein synthesis pathways. GC-related lipid metabolism abnormalities lead to coordinated changes in adipokines such as leptin and adiponectin, resulting in bone remodeling imbalance, muscle protein breakdown, and muscle mass loss. Concurrently, GC-induced insulin resistance and decreased IGF-1 levels also simultaneously impair the ability of osteoblasts and myocytes to utilize glucose and fatty acids, causing both bone and muscle ends to operate under the same metabolic pattern. Consequently, a multi-layered shared lipid metabolism signaling network is constructed between the bone and muscle ends, forming the metabolic basis for bone-muscle energy imbalance.

Current studies on the lipid metabolism axis in SONFH have mainly focused on BMSC adipogenic shift and intramuscular lipid accumulation, yet several key questions remain unanswered. First, most studies have explored the roles of individual adipokines (e.g., leptin, adiponectin) in bone or muscle injury separately, lacking a systematic analysis of adipokine-mediated crosstalk between the bone marrow and skeletal muscle microenvironments under GC exposure (91). Second, the specific threshold of lipid overload that triggers irreversible bone-muscle synergistic damage has not been defined, and there is a paucity of longitudinal studies tracking the dynamic changes in systemic and local lipid metabolism during the progression from early GC exposure to symptomatic SONFH (92). Furthermore, whether lipid metabolism disorders in the bone-muscle unit are a primary systemic effect of GCs or a local secondary change following ischemic injury remains controversial, necessitating further verification through tissue-specific conditional knockout models.

## 4 Activation of the inflammation-immune axis: sustained bone-muscle injury

### 4.1 Inflammatory-immune response in bone tissue induced by GCs

In SONFH, the epiphyseal bone exhibits a focal inflammatory-immune response characterized by low-grade, persistent inflammatory activation (93, 94) and disruption of immune homeostasis (95). On one hand, GCs exert systemic anti-inflammatory effects by suppressing the proliferation and function of immune cells (96). On the other hand, within the context of bone marrow adiposity, impaired microcirculatory perfusion, and increased osteocyte apoptosis, damage-associated molecular patterns (DAMPs) are released from the damaged bone tissue (97), including high mobility group box 1 (HMGB1) and ATP (98). Necrotic osteocytes can regulate osteoclastogenesis through the interaction between HMGB1 released from dead bone cells and pattern recognition receptors (PRRs) on osteoclast precursors (97). DAMPs released from necrotic osteocytes can also bind to PRRs, such as TLR2, TLR4, and RAGE, on immune cells including macrophages and dendritic cells, promoting their production of pro-inflammatory cytokines like TNF-α and IL-6. These cytokines further induce RANKL expression in osteoblast lineage cells or directly promote osteoclastogenesis (99). This inflammatory-immune response mechanism systematically affects the homeostasis of the bone marrow microenvironment, creating an imbalance between local pro-inflammation and systemic anti-inflammation, which contributes to long-term bone tissue injury.

**Figure 5:**
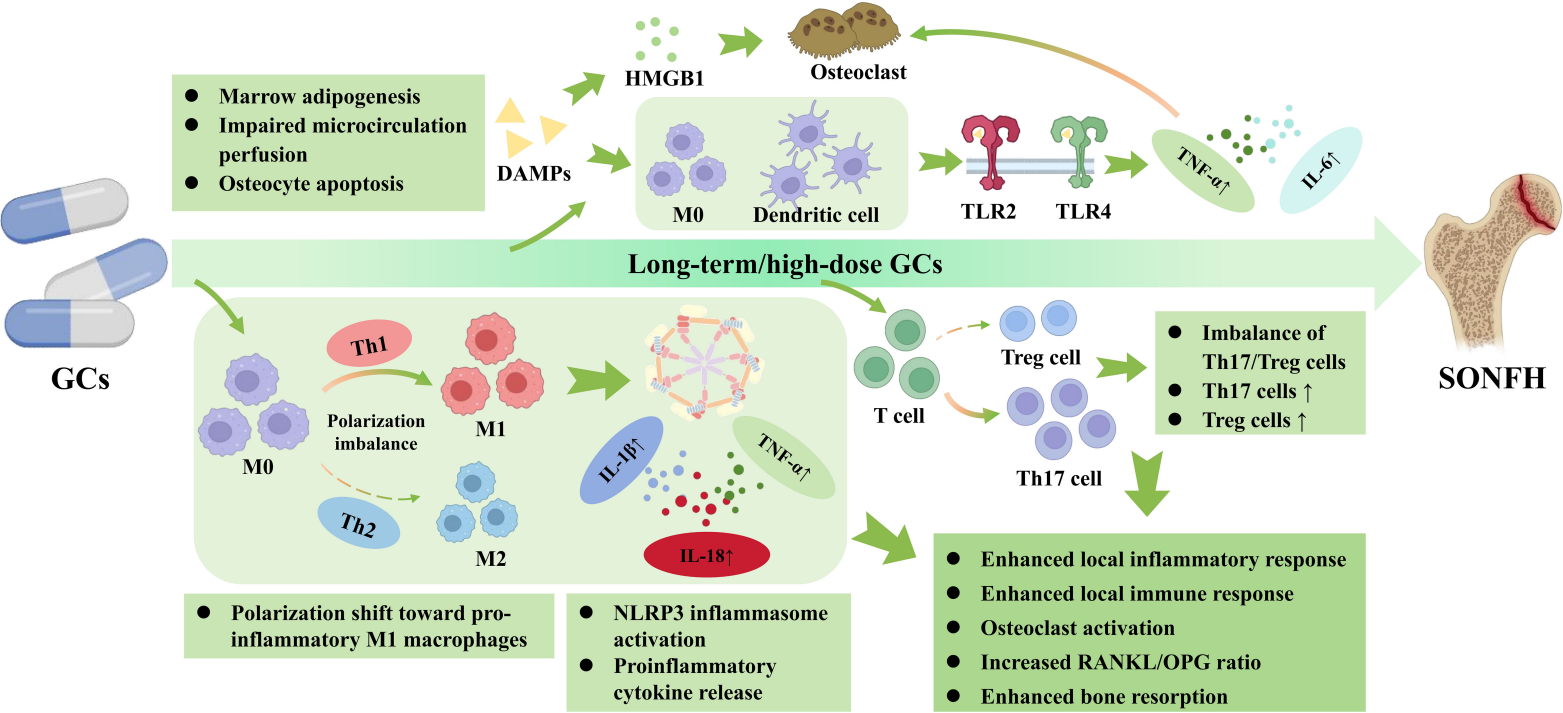
The pathological mechanisms of the inflammation-immune axis within the bone microenvironment. GCs trigger an immune imbalance characterized by a polarization shift toward pro-inflammatory M1 macrophages and a pathologically increased Th17/Treg cell ratio. These dysregulated immune cells, combined with the activation of the NLRP3 inflammasome, release abundant pro-inflammatory cytokines (e.g., IL-1β, TNF-α, and IL-6). Binding to specific receptors (e.g., TLR2/TLR4), these cytokines create an enhanced local inflammatory response that markedly elevates the RANKL/OPG ratio, thereby overactivating osteoclasts and driving rapid bone resorption in SONFH. Solid arrows denote direct molecular activation or pathogenic progression. Upward and downward arrows (↑/↓) indicate upregulation or downregulation.

Activated by epiphyseal macrophages and bone marrow endothelial cells, the polarization of local immune cells in the bone marrow shifts towards pro-inflammatory M1-type macrophages (100), accompanied by activation of the NLRP3 inflammasome, which in turn promotes the sustained release of pro-inflammatory cytokines such as IL-1β, IL-18, and TNF-α (95, 101). IL-1β and TNF-α can further amplify the local inflammatory response by activating signaling pathways like NF-κB and MAPK, promoting osteoclast activation and bone resorption (102, 103). Concurrently, the RANKL/osteoprotegerin (OPG) ratio increases in epiphyseal osteoblasts and bone marrow stromal cells (104, 105), indicating an enhanced stimulatory effect of RANKL on osteoclast precursors, thereby augmenting osteoclast generation and bone resorptive activity. Furthermore, under long-term GCs exposure, the bone marrow T-cell lineage undergoes remodeling, and the imbalance between Th17 and Treg cells is considered a key factor in this process (106). Th17 cells activate osteoclast function by secreting cytokines such as IL-17, further enhancing RANKL expression and exacerbating bone resorption. The reduction in Treg cells leads to a loss of immune tolerance, intensifying the local immune response. The imbalance in bone remodeling caused by the aforementioned inflammatory-immune abnormalities exacerbates the destruction of trabecular microstructure, leading to a gradual loss of the biomechanical support capacity of the femoral head, thereby potentially accelerating the process of femoral head collapse (**Figure 5**).

### 4.2 Muscle inflammation-immune response induced by glucocorticoids

Long-term or high-dose GCs exposure alters fatty acid metabolism in muscle, leading to the accumulation of fatty acids within muscle tissue. Excessive fatty acid accumulation not only results in incomplete fatty acid oxidation but also activates inflammatory transcription factors such as NF-κB (107, 108). This subsequently promotes the expression of myogenic inflammatory factors, including interleukin-6 and TNF−α, by muscle fibers and interstitial cells. Concurrently, studies have demonstrated that inflammatory cytokines such as TNF−α and interferon-gamma (IFNγ) can induce muscle atrophy via signal transducer and activator of transcription 3 (STAT3) activation (109). These inflammatory factors recruit macrophages through local signaling and promote their polarization towards a pro-inflammatory M1 phenotype. M1 macrophages further release pro-inflammatory cytokines (101), establishing a vicious cycle. Furthermore, GCs act directly on muscle cells via the glucocorticoid receptor, activating the key inflammatory signaling pathway involving Forkhead box O (FoxO) transcription factors. This drives the expression of muscle-specific E3 ubiquitin ligases, such as muscle RING-finger protein-1 (MuRF1) and muscle atrophy F-box/Atrogin-1 (110, 111). These enzymes promote the ubiquitin-proteasome-mediated degradation of muscle proteins, thereby further promoting muscle proteolysis. This process is intricately linked to the inflammatory response, forming a positive feedback loop between inflammation and atrophy. Specifically, inflammatory cytokines upregulate MuRF1 and Atrogin-1, enhancing muscle protein degradation. The resulting degradation products can, in turn, exacerbate local inflammation, further accelerating muscle wasting.

**Figure 6:**
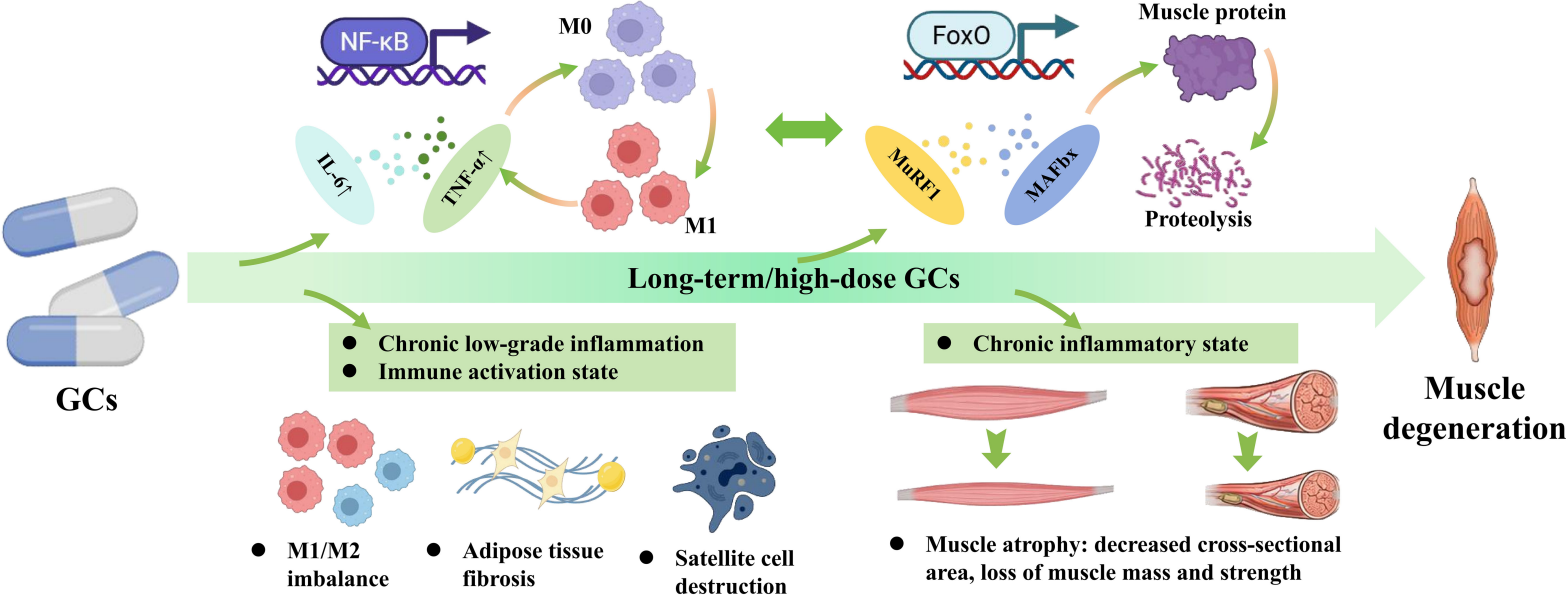
The pathological mechanisms of the inflammation-immune axis within the muscle microenvironment. GCs induce a state of chronic low-grade inflammation, distinctly marked by M1/M2 macrophage imbalance and elevated circulating cytokines (such as TNF-α and IL-6). This chronic inflammatory state continuously activates the NF-κB and FoxO signaling pathways, which in turn strongly upregulate muscle-specific E3 ubiquitin ligases (MuRF1 and MAFbx/Atrogin-1). The robust activation of these ligases executes rapid proteolysis, leading to substantial muscle protein loss, decreased cross-sectional area, and definitive muscle degeneration. Solid arrows indicate direct molecular activation or pathological progression. Upward and downward arrows (↑/↓) denote upregulation or downregulation.

When muscle is in a state of chronic low-grade inflammation or immune activation, its regenerative capacity is significantly impaired. Chronic inflammation inhibits the activation, proliferation, and differentiation of satellite cells by altering macrophage polarization (M1/M2 imbalance) within muscle tissue (103), promoting adipofibrosis (112), and disrupting the satellite cell niche (113). This leads to poor regeneration, muscle atrophy, and functional loss. Simultaneously, a persistent inflammatory state induces a shift in muscle fiber type composition, with particularly pronounced atrophy of type II fast-twitch fibers (114). This is accompanied by a reduction in fiber cross-sectional area and a loss of muscle mass and strength (115). More importantly, the chronic low-grade inflammation induced by GCs not only directly damages muscle structure and function but also exacerbates the pathological progression of SONFH by disrupting the muscle-bone axis (**Figure 6**).

### 4.3 Shared pathways and axial linkages in bone-muscle inflammatory-immune responses

In SONFH, the inflammatory–immune responses in bone and muscle do not occur in isolation. Instead, they are interconnected through multiple shared signaling pathways, forming a synergistically amplified bone-muscle inflammatory-immune axis. These shared immune pathways transmit signals between bone and muscle, exacerbating local immune responses and further driving the occurrence and progression of SONFH.

The NF-κB and STAT3 pathways, serving as shared inflammatory transcription factors in bone and muscle, play a crucial role under GC exposure. In bone, GCs activate the NF-κB pathway, promoting the release of pro-inflammatory cytokines such as TNF-α and IL-1β, thereby enhancing bone resorption. In muscle, NF-κB is also activated, promoting muscle protein degradation, particularly through the upregulation of ubiquitin ligases like MuRF1 and MAFbx/Atrogin-1, leading to muscle atrophy. In bone, STAT3 promotes osteoclastogenesis by enhancing RANKL expression, while in muscle, STAT3 amplifies the inflammatory response by regulating cytokines such as IL-6. These intertwined shared signaling pathways lead to the synchronous enhancement of inflammation and immune responses at both bone and muscle sites. Secondly, Th17/Treg imbalance is another key factor in the bone-muscle inflammatory response. Th17 cells promote osteoclastogenesis by secreting cytokines such as IL-17. In muscle, upregulated IL-17 similarly promotes the expression of muscle atrophy factors by activating the NF-κB pathway, further exacerbating muscle wasting. Concurrently, the reduction in Treg cells leads to a loss of immune tolerance, intensifying local inflammatory responses and making the immune reactions in both bone and muscle more severe. Furthermore, the interaction between immune cells in bone and muscle further exacerbates the inflammatory response. Both bone marrow-derived macrophages and intramuscular macrophages exhibit M1 polarization under GC influence. The former promotes bone resorption by secreting pro-inflammatory cytokines, while the latter enhances muscle atrophy and inflammation, establishing a pathological state where bone resorption and muscle atrophy advance in parallel.

It is important to acknowledge the current evidentiary boundaries regarding these immune mechanisms. While the isolated pathogenic roles of the NLRP3 inflammasome and Th17/Treg imbalance are increasingly recognized in glucocorticoid-induced osteonecrosis and myopathy independently, direct *in vivo* evidence capturing their simultaneous, cross-tissue crosstalk specifically within the SONFH bone-muscle unit remains limited. Consequently, our understanding of this integrated inflammatory-immune signal transmission is partially extrapolated from mechanistically adjacent musculoskeletal comorbidities, such as glucocorticoid-induced osteoporosis and osteoarthritis. Rather than diminishing the relevance of these pathways, this extrapolation highlights a critical theoretical frontier. These inflammatory cascades represent highly probable, yet underexplored, molecular bridges in SONFH pathogenesis, thereby underscoring the urgent need for targeted validation in future multi-axis research.

Although shared inflammatory pathways in bone and muscle injuries have been extensively reported (116), an in-depth exploration of the immune axis within the context of SONFH-related bone-muscle crosstalk remains insufficient. First, current studies are predominantly limited to the roles of macrophages and common pro-inflammatory cytokines (95, 100), while the contributions of other immune cell subsets (such as innate lymphoid cells and B cells) and specialized pro-resolving mediators (117) within the bone-muscle unit remain largely uncharacterized. Second, the spatiotemporal characteristics of the immune response in the bone-muscle unit during SONFH progression have yet to be elucidated; specifically, it remains unclear whether the inflammatory response originates in the bone marrow or the peri-hip muscle, and how immune signals are transduced between these two tissues. Third, a lack of evidence from tissue-specific immune cell knockout models persists to verify the causal role of immune imbalance in bone-muscle co-damage (118), making it challenging to distinguish primary immune responses from secondary changes throughout the disease course.

## 5 Mechanical axis abnormality: bone-muscle synergistic instability

### 5.1 Interdependence and instability of the bone-muscle mechanical axis

Within the multi-axis pathological model of SONFH, the mechanical axis constitutes a crucial yet historically under investigated dimension in the pathological research of SONFH. Muscles generate tension through contraction, which is transmitted via tendons to bones, thereby applying mechanical loads to bone structures. The adaptive response of bone to such loading is one of the vital mechanism for maintaining bone mass and strength (22, 119). If GCs use leads to muscle dysfunction, the mechanical stimulation to bone is reduced. This may trigger an imbalance in bone remodeling (where bone resorption exceeds bone formation) (120, 121), which has been associated with osteocyte death, trabecular bone disruption, and osteoporosis (122), which subsequently contributes to the onset and progression of SONFH. Therefore, the mechanical transmission and alterations in the mechanical environment between bone and muscle should be regarded as a key component of the multi-axis pathological model of SONFH.

As a pivotal joint connecting the trunk and lower limbs, the normal biomechanical function of the hip relies on the close coordination between bone and muscle/soft tissues (123). The skeletal structure (e.g., the femoral head–acetabulum complex) provides rigid support for the joint, while muscles, through contraction and relaxation, tendon force transmission, and the synergistic action of soft tissues (joint capsule, labrum, ligaments), undertake functions such as load sharing, mechanical stabilization, and load regulation (124, 125). In this process, bone and muscle work together to maintain hip joint stability, preventing excessive movement of the femoral head and potential collapse risks. Under healthy conditions, periarticular hip muscles (e.g., gluteus maximus, hip flexors, and quadriceps) play a crucial supportive role by stabilizing the femoral head’s position and reducing joint wear. Under normal mechanical conditions, forces generated by muscles are evenly distributed across the femoral head, while trabecular bone internally shares and bears stress, establishing a state of mechanical equilibrium. Recent finite element and stress trajectory analyses have confirmed that changes in muscle forces can significantly affect the strain/stress distribution in the proximal femur (126). The morphology and orientation of trabecular bone are highly consistent with the principal stress directions (127), indicating that its structure is an adaptation to normal mechanical loading conditions. The biomechanical properties of cancellous bone make it a key component for dispersing and withstanding mechanical loads (128). The latest research also emphasizes the important structural role of trabecular bone in hip joint stability and load distribution (129).

However, in SONFH, this bone-muscle mechanical coupling is disrupted. Due to decreased bone density and degradation of bone microstructure induced by GC exposure, the mechanical load-bearing capacity of the bone is reduced (130), increasing the risk of collapse. Concurrently, muscle atrophy and functional loss prevent effective mitigation of the loads borne by the bone. This leads to increased concentrated stress on the bone ends (131), ultimately causing bone collapse under uneven stress. Consequently, the interdependence between bone and muscle is progressively broken down. External forces acting on the femoral head cannot be evenly distributed, resulting in mechanical axis instability. This creates conditions conducive to femoral head collapse and the occurrence of osteonecrosis.

### 5.2 Impact of GCs on the imbalance of the bone-muscle mechanical axis

**Figure 7:**
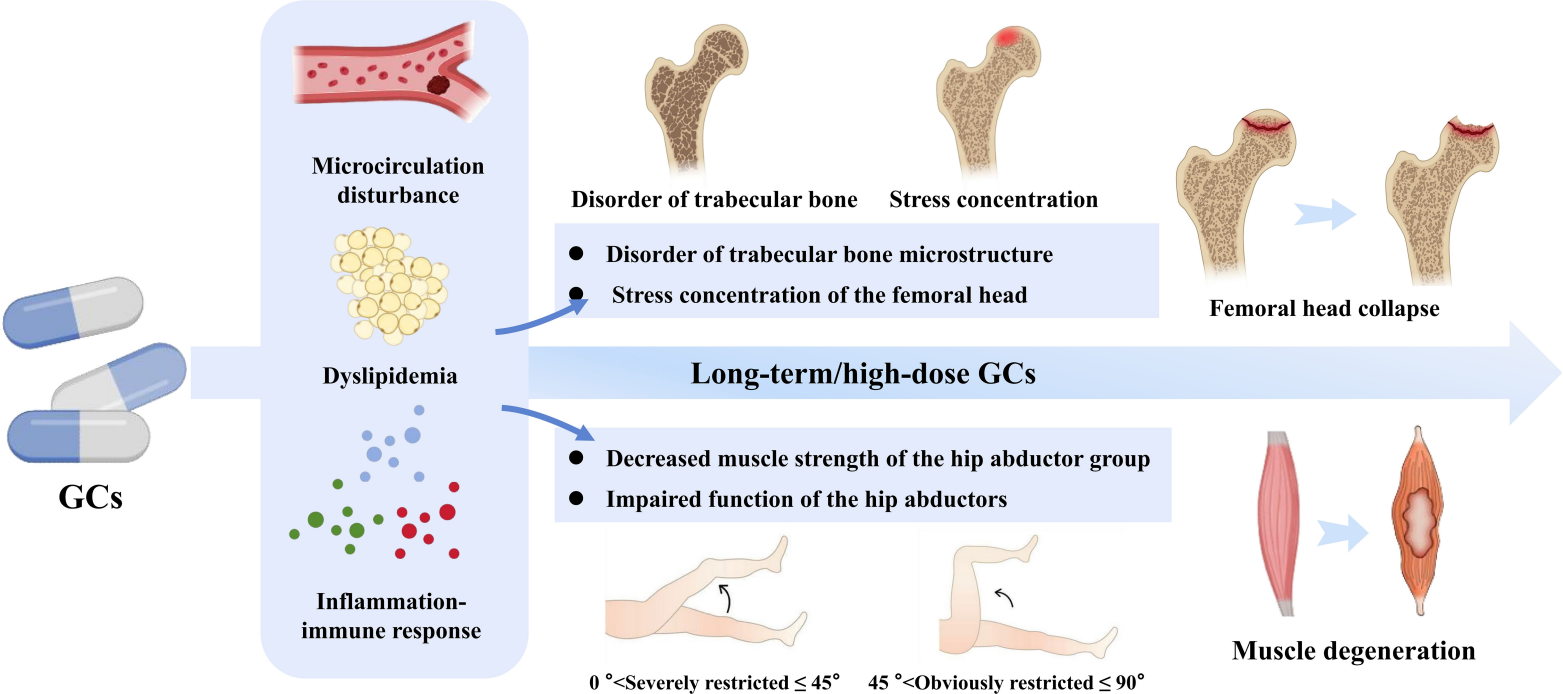
The pathological mechanisms of the mechanical axis within the bone-muscle unit. GCs induce a triad of profound upstream disruptions: microcirculation disturbances, dyslipidemia, and severe inflammation-immune responses. These preceding systemic alterations convergently assault the mechanical load-bearing and dynamic supportive capacities of the hip joint. Within the osseous structures, this massive pathological cascade provokes severe disorders of the trabecular bone microstructure, leading to focal stress concentration and the inevitable structural collapse of the femoral head. Concurrently, within the muscular system, these systemic insults severely decrease the muscle strength of the hip abductor group and impair dynamic functional mobility (clinically manifesting as severely restricted hip abduction angles), ultimately driving macroscopic muscle degeneration. This synergistic deterioration creates a definitive biomechanical breakdown, highlighting the simultaneous structural instability and functional loss inherent in SONFH. Solid arrows indicate the overarching progression of macro-level pathological events and the sequential trajectory of structural and functional collapse.

The influence of GCs on the bone-muscle mechanical axis in SONFH is comprehensive, involving the synergistic effects of multiple pathways such as microcirculatory homeostasis, lipid metabolism balance, and inflammatory-immune responses, which subsequently alter the mechanical support between bone and muscle. Impairment of femoral head microcirculation (29), excessive accumulation of intraosseous fat (67), and persistent inflammatory-immune responses (93–95) lead to the destruction of trabecular microstructure, increase the fragility of the femoral head, prevent effective dispersion of the load it bears, significantly reduce the load-bearing capacity of bone tissue, and result in localized stress concentration, thereby further elevating the risk of femoral head collapse. Concurrently, muscle atrophy induced by GC exposure (124) further contributes to mechanical instability (132). Insufficient muscle strength reduces the mechanical loading on the skeleton (133), with the hip abductor muscles being particularly crucial for femoral head stability (13, 134). Under normal biomechanical conditions, the hip abductor muscles generate muscular moments to counteract the medial moment caused by body weight, thereby regulating hip joint contact forces and the distribution of load on the femoral head (135). However, muscle atrophy under GC exposure compromises this regulatory mechanism, exacerbates localized stress on the femoral head, and consequently potentially accelerating the destruction of bone structure and the occurrence of femoral head necrosis and collapse (**Figure 7**).

### 5.3 Mechanical axis imbalance and the vicious cycle of bone-muscle co-damage

The stress distribution within the femoral head is highly dependent on the integrity of the trabecular bone and the dynamic stability provided by the periarticular hip muscles. With the dual impact of GCs on both bone and muscle, a self-perpetuating cycle of mechanical axis imbalance gradually forms: bone damage increases the load burden on muscles, while muscle atrophy exacerbates bone damage. During the progression of SONFH, the yield strength of the trabecular structure in the necrotic area decreases, leading to stress concentration in the weight-bearing dome region (the curved area in the anterolateral part of the femoral head), which becomes a critical weak point for collapse (136). Following the onset of osteonecrosis, a decline in muscle strength of the periarticular hip muscles occurs due to factors such as ischemia, inflammation, and fatty infiltration (13), further compromising joint stability and leading to overall mechanical instability of the joint (137). This establishes a vicious cycle of bone damage → muscle atrophy → bone damage. This vicious cycle not only accelerates the process of bone-muscle damage but also hastens the occurrence of femoral head collapse, further contributing to the loss of joint stability and rapid functional decline. Therefore, mechanical axis imbalance represents not merely the functional loss of a single tissue but rather a dual impairment characterized by the mutual influence and constraint between bone and muscle. This necessitates that the treatment of osteonecrosis of the femoral head considers the characteristics of bone-muscle co-damage, intervening in both bone and muscle from a holistic perspective to prevent further aggravation of this vicious cycle.

Current research on the mechanical axis in SONFH predominantly focuses on finite element analysis of the femoral head and cross-sectional clinical observations of muscle atrophy, leaving the mechanistic exploration of mechanical signal-mediated bone-muscle crosstalk in its infancy (138). Although most studies describe a correlation between muscle atrophy and femoral head collapse (139), the causal relationship between reduced mechanical loading and bone-muscle synergistic degeneration remains inadequately verified by *in vivo* mechanical intervention studies. Furthermore, the specific mechanotransduction pathways (such as YAP/TAZ, Piezo1) (140, 141) transmitting mechanical signals between bone and muscle have not been systematically elucidated in the context of SONFH. Consequently, this knowledge gap significantly limits the translation of basic research findings into clinical risk stratification and therapeutic efficacy evaluation.

## 6 Interactive amplification effects of four pathological axes: the comprehensive pathological mechanism of SONFH

**Figure 8:**
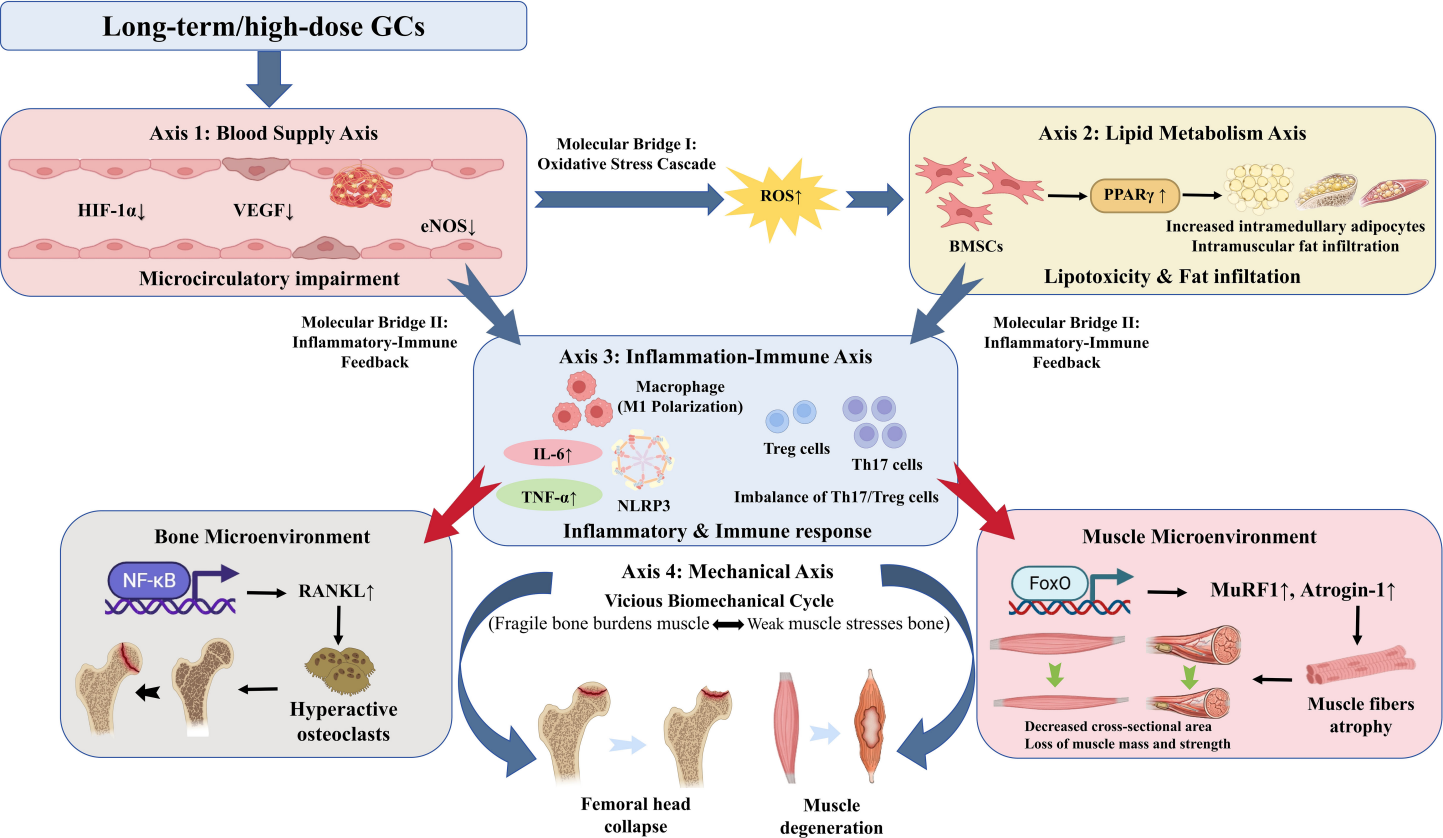
Integrated schematic diagram of the synergistic amplification network of bone-muscle crosstalk in SONFH driven by critical molecular bridges. GCs initiate the pathological cascade by disrupting the Blood Supply Axis (Axis 1), leading to microcirculatory impairment via the downregulation of HIF-1α, VEGF, and eNOS. This severe ischemia acts as an amplifying engine, triggering Molecular Bridge I (Oxidative Stress Cascade), where the overproduction of ROS activates PPARγ within the Lipid Metabolism Axis (Axis 2). This induces severe lipotoxicity, accelerating BMSC adipogenesis and causing widespread fat infiltration in both bone and muscle tissues. Convergently, ischemic and lipotoxic damage triggers Molecular Bridge II (Inflammatory-Immune Feedback), driving macrophage M1 polarization and Th17/Treg cell imbalance within the Inflammation-Immune Axis (Axis 3). The resulting release of key pro-inflammatory cytokines, notably TNF-α and IL-6, acts as a powerful dual-tissue disruptor. Within the bone microenvironment, these cytokines upregulate RANKL via NF-κB signaling, inducing osteoclast hyperactivity. Concurrently, within the muscle microenvironment, they strongly upregulate MuRF1 and Atrogin-1 via FoxO signaling, executing severe myofibrillar atrophy. Ultimately, this simultaneous destruction converges on the Mechanical Axis (Axis 4), initiating a vicious biomechanical cycle where fragile bone and weakened muscle mutually exacerbate stress, inevitably precipitating the final structural collapse of the femoral head and macroscopic muscle degeneration. Thick arrows indicate the overarching multi-axis sequential progression and inter-axis molecular bridges. Thin solid arrows denote intra-axis molecular activation or pathological pathways. Upward and downward arrows (↑/↓) denote the pathological upregulation or downregulation of specific targets, respectively.

SONFH is a complex progressive disease involving the interaction of multiple pathological axes. The vascular supply axis, lipid metabolism axis, inflammation-immune axis, and mechanical axis are intricately intertwined through shared signaling pathways and cross-tissue mediators, forming a synergistic and exacerbating pathological network that collectively drives bone-muscle co-damage and disease progression. Each axis not only independently affects the structure and function of bone and muscle but also amplifies the damage of the others through their interactions, jointly leading to a bone-muscle comorbidity (**Figure 8**).

However, current research on multi-axis interaction in SONFH still has significant limitations: most studies have only described the parallel changes of merely two isolated pathological axes (e.g., lipid metabolism and inflammation, blood supply and mechanical load), lacking a systematic analysis of the four-axis synergistic network within the same disease model. Specifically, the precise hierarchical and sequential relationships among the four axes havs not been fully elucidated by dynamic *in vivo* studies. Rather than a strictly linear progression, Early GC exposure likely triggers a parallel activation of multiple axes due to the widespread distribution of glucocorticoid receptors. Within this dynamic spatiotemporal model, the blood supply axis functions as the central “amplifying axis”, serving as a critical catalyst that drives the synergistic deterioration of the entire network. Furthermore, the key “hub molecules” that simultaneously regulate these multiple axes remain to be identified, representing potential core targets for the concurrent intervention of bone-muscle co-damage.

To better address the molecular dynamics of SONFH, it is essential to delineate the dominant mechanisms across the various stages of the disease. Although these four axes are activated in parallel rather than in a strictly linear sequence, their pathological contributions shift progressively:

(1) Early Stage: During the initial phase of GC exposure, the pathogenesis is predominantly driven by the blood supply and lipid metabolism axes, accompanied by early inflammatory priming. GCs induce microcirculatory impairment (via inhibition of HIF-1α/VEGF and eNOS) and direct BMSCs toward adipogenesis (via upregulation of PPARγ). Crucially, the resultant hypoxia, lipotoxicity, and ROS overproduction initiate a persistent low-grade inflammatory response. At this stage, while bone and muscle structures remain macroscopically intact, their microenvironments are fundamentally altered by this parallel ischemic, metabolic, and early inflammatory stress. (2) Intermediate Stage: As the disease progresses, the inflammation-immune axis transitions from early priming to massive amplification, acting as the primary driver of tissue destruction. The continuous release of DAMPs and adipokines (e.g., leptin) from ischemic and fatty tissues exacerbates M1 macrophage polarization and Th17/Treg imbalance. The robust secretion of pro-inflammatory cytokines (such as TNF-α and IL-6) functions as a dual-tissue disruptor. In bone, these cytokines strongly upregulate RANKL via NF-κB and STAT3 signaling, accelerating osteoclast-mediated bone resorption. Simultaneously in muscle, they upregulate E3 ubiquitin ligases (MuRF1 and Atrogin-1) via FoxO signaling, executing rapid myofibrillar degradation. (3) Late Stage: In the advanced stage, the cumulative structural damage converges on the mechanical axis. The severely resorbed trabecular bone loses its load-bearing capacity, while the atrophied periarticular hip muscles fail to provide dynamic stabilization. This mechanical uncoupling creates a terminal vicious cycle: weakened bone increases the biomechanical burden on failing muscles, while atrophied muscles exacerbate localized stress concentration on the fragile femoral head. Ultimately, this uneven stress distribution precipitates irreversible macroscopic collapse and severe functional disability.

The amplification effect of the blood supply axis is profoundly evident in its interaction with the lipid metabolism axis. GC-induced vascular impairment, driven by the inhibition of endothelial cell function and reduction in vasodilator production, leads to decreased local microcirculatory perfusion. This insufficient blood supply catalyzes a severe hypoxic microenvironment that further aggravates ischemia by promoting bone marrow adiposity. At the molecular level, this sustained ischemia triggers mitochondrial dysfunction and the overproduction of ROS. This hypoxic and ROS-rich environment acts as a critical molecular bridge, directly upregulating the expression of PPARγ while simultaneously suppressing osteogenic signaling pathways. This transcriptional redirection forcibly shunts BMSCs toward adipogenesis rather than osteogenesis. The resulting accumulation of adipocytes not only physically reduces blood flow by compressing blood microvessels but also interferes with fatty acid utilization by bone cells via the secretion of adipokines, thereby weakening the metabolic and reparative capacity of bone tissue. Consequently, impaired perfusion and lipid metabolic disorders form a primary vicious cycle, mutually amplifying local ischemia and accelerating the progression of femoral head necrosis.

Driven by this early metabolic and microcirculatory stress, the interaction between the lipid metabolism axis and the inflammation-immune axis becomes particularly pronounced. Abnormal lipid metabolism triggers a chronic low-grade inflammatory response by activating adipokines such as leptin and adiponectin, which exacerbating the imbalance in bone remodeling and promotes bone resorption. Additionally, these adipokines amplify local inflammatory responses and accelerate the process of bone marrow adiposity by disrupting the immune homeostasis in both bone and muscle. This intersection of lipid metabolic disorders and immune activation constitute a severe double hit, leading not only to accelerated bone loss but also to exacerbated muscle decline and atrophy.

Ultimately, the synergistic destruction caused by ischemia, lipotoxicity, and immune activation converges on the mechanical axis, precipitating final structural failure. This critical uncoupling is mediated by specific molecular bridges, predominantly inflammatory cytokines such as TNF-α and IL-6, which function as dual-tissue disruptors. GC-induced vascular and metabolic impairments directly compromise the mechanical load-bearing capacity of bone. Specifically, within the osseous microenvironment, TNF-α and IL-6 activate the NF-κB and STAT3 signaling pathways to strongly upregulate RANKL expression, accelerating osteoclast-mediated bone resorption. The resulting destruction of trabeculae and the decrease in bone density result in the femoral head’s inability to effectively distribute and withstand loads, increasing local stress concentration and initiating mechanical axis instability. Concurrently, ischemia and inflammation-driven muscle atrophy further weakens hip joint stability. At the molecular level, these same circulating inflammatory factors activate the STAT3 and FoxO/NF-κB pathways within the muscular microenvironment. This robustly drives the expression of muscle-specific E3 ubiquitin ligases, notably MuRF1 and Atrogin-1, executing rapid myofibrillar degradation. The decline in strength of the periarticular hip muscles deprives the femoral head of essential dynamic support during weight-bearing. This breakdown creates a definitive, mutually reinforcing situation: weakened bone increases the biomechanical burden on atrophied muscles, while failing muscles exacerbate the localized stress on the fragile bone, making the femoral head highly susceptible to complete collapse.

## 7 Future perspectives

SONFH is a progressive musculoskeletal disease with complex etiology and incompletely understood pathophysiology. Particularly for young patients, an optimal treatment strategy is currently lacking. Although the “four-axis framework of bone-muscle crosstalk” proposed in this review systematically integrates the scattered pathological findings of SONFH, current research still has several key gaps that need to be addressed in future studies.

### 7.1 Elucidating the multi-axis interactive network and hierarchical sequential relationship of bone-muscle crosstalk in SONFH

Current studies are largely confined to exploring the independent roles of single pathological axes in either bone or muscle injury, leaving the complex interplay among these four axes largely uncharacterized. At the molecular and multi-omics level, there is a critical need for direct validation of the four-axis-mediated crosstalk between bone and muscle in SONFH using multidimensional technologies. For instance, spatial multi-omics (142, 143) could be employed to map the dynamic molecular changes within the hip bone-muscle unit across different stages of SONFH. Specifically, by integrating spatial transcriptomics with mass spectrometry imaging, the “molecular bridges” (e.g., specific lipid metabolites or inflammatory cytokines) can be precisely localized at the tissue interface between bone and muscle, thereby identifying the key hub molecules that orchestrate the synergistic amplification of these four axes. Building upon this, at the *in vivo* level, the causal relationships of these axes must be rigorously verified using tissue-specific conditional knockout or transgenic animal models (144) (e.g., endothelial cell-specific HIF-1α knockout or myocyte-specific PPARγ overexpression). To establish definitive causality, a “cross-tissue intervention” design is essential: for example, utilizing the Cre-LoxP system (e.g., Ocn-Cre for osteoblasts and Myf5-Cre for myocytes) to selectively knockout a hub molecule in bone tissue and observing its secondary pathological impact on muscle function, or vice-versa.

Concurrently, at the *in vitro* cellular level, complex microfluidic organ-on-a-chip systems should be established to simulate the microenvironment of the bone-muscle unit. This setup incorporates fluid shear stress and oxygen gradients to better mimic the ischemic-hypoxic conditions of SONFH, allowing for the direct observation of cross-tissue pathological signal transmission among the four axes under GC intervention.

### 7.2 Developing combined intervention strategies targeting the bone-muscle unit and multi-pathological axes

Current interventional studies for SONFH predominantly target a single pathological mechanism (29) (e.g., pro-angiogenic therapies, anti-inflammatory drugs, or lipid-lowering agents) or focus exclusively on bone structural repair. Consequently, few interventions address the concomitant bone-muscle comorbidity and the synergistic multi-axis injury. To address this, at the pharmacological level, there is an urgent need for preclinical studies to verify the efficacy of synergistic drug combinations targeting multiple pathological axes. For instance, the combined application of bone morphogenetic proteins and angiogenic growth factors (145), or lipid metabolism regulators with mechanotransduction modulators (146), could be evaluated to rigorously compare their therapeutic effects on bone-muscle comorbidity and disease progression against conventional single-target therapies. Furthermore, at the translational delivery level, the development of advanced targeted delivery systems (e.g., bone-muscle dual-targeted nanomedicines) capable of simultaneously modulating multiple pathological axes represents a highly promising therapeutic strategy. For instance, Yang et al. developed a dual-targeted approach to simultaneously promote the repair of damaged vascular endothelium and the re-modulation of BMSCs, evaluating its efficacy in halting the progression of SONFH (147). Similarly, Zhao et al. innovatively employed nanoclusters to directly target and modulate the muscle-bone metabolic axis (148).

### 7.3 Establishing a clinical evaluation system integrating bone-muscle unit function and multi-axis biomarkers

Currently, the clinical diagnosis and risk stratification of SONFH rely predominantly on radiographic changes within the femoral head, lacking a systematic evaluation of peri-hip muscle status and the underlying pathological alterations across the four interconnected axes. Future research should prioritize the following two translational directions: In terms of diagnostic framework optimization, there is a critical need to establish a standardized, quantitative evaluation system for the hip bone-muscle unit that refines current SONFH staging diagnoses (e.g., the ARCO staging). This comprehensive system should explicitly incorporate multidimensional metrics into clinical staging, including imaging indicators (e.g., muscle cross-sectional area and the rate of peri-hip muscle fat infiltration via MRI), specific bone-muscle mechanical coupling parameters (e.g., hip muscle strength and gait analysis), and circulating biomarkers representative of the four axes. Implementing such a framework will enable early screening and accurate risk stratification of SONFH well before the onset of irreversible radiographic collapse. Regarding longitudinal clinical validation, by integrating serial blood sampling for multi-omics profiling with the aforementioned multidimensional clinical metrics, researchers can identify “early-warning” molecular signatures and verify whether dynamic changes in bone-muscle unit function can reliably predict the progression of SONFH and the clinical efficacy of joint-preserving treatments. Ultimately, establishing these predictive models will provide objective, evidence-based indicators to guide personalized therapeutic decision-making.

Although the four-axis framework of bone-muscle crosstalk proposed in this study systematically integrates the existing pathological findings of SONFH, the inherent limitations of this review should be objectively acknowledged. The integrated pathophysiological model constructed herein is primarily synthesized from independent studies focusing on a single tissue, either bone or muscle. Therefore, it still needs to be rigorously verified through well-designed experimental and clinical studies. Future research must employ longitudinal clinical cohorts and cross-tissue intervention models to clarify the definitive causal relationships among the four axes and validate their comprehensive effects on the progression of SONFH.
